# Elevated preoperative neutrophil/lymphocyte ratio as a predictor of increased long-term survival in minimal invasive coronary artery bypass surgery compared to sternotomy

**DOI:** 10.1186/1749-8090-8-193

**Published:** 2013-09-27

**Authors:** Basem Azab, Masood A Shariff, Rana Bachir, John P Nabagiez, Joseph T McGinn

**Affiliations:** 1 Department of Surgery, Staten Island University Hospital, 475 Seaview Ave, Staten Island, NY, USA; 2 Cardiothoracic Surgery Department, Staten Island University Hospital, 475 Seaview Ave, Staten Island, NY, USA

**Keywords:** Inflammatory cells, Minimally invasive surgery, CABG, Thoracotomy, Outcomes

## Abstract

**Background:**

Neutrophil lymphocyte ratio (NLR) is a predictor of major adverse cardiovascular outcomes. Our study explores the value of NLR in predicting long-term mortality after minimally invasive coronary artery bypass surgery (MICS) via lateral left-thoracotomy versus conventional sternotomy coronary artery bypass grafting (CABG) surgery.

**Methods:**

A total of 1126 consecutive patients (729 sternotomy CABG and 397 MICS) from a single tertiary center between 2005 and 2008 were followed until 2011. We stratified the patients into equal tertiles according to preoperative NLR. The primary outcome, all-cause mortality, was compared among the NLR tertiles.

**Results:**

Out of the 1126 patients included in the study, 1030 (91%) patients underwent off-pump CABG . The first (NLR <2.3) tertile had a significantly lower 5-year mortality (30/371 =8%) in comparison to the second (NLR =2.3-3.4) and third (NLR ≥3.5) tertiles (49/375 =13% and 75/380 =20%), respectively with p < 0.0001. After multivariate adjustment, NLR was a significant independent predictor of mortality (hazard ratio [HR] per each unit increase of NLR was 1.05, 95% confidence interval [CI] 1.01-1.10, p = 0.008). MICS and sternotomy CABG groups with NLR <3 had similar mortality (21/221 =9.5% and 40/403 =9.9%), p = 1. However among patients with NLR ≥3, MICS had a significantly lower mortality (23/176 = 13.1%) compared to the sternotomy CABG (70/326 =21.5%), p = 0.02. According to the multivariate analysis of patients with NLR ≥3, MICS had a significantly lower mortality compared to sternotomy CABG (HR = 0.44, 95% CI 0.24-0.78, p = 0.005).

**Conclusion:**

Elevated preoperative NLR is an independent predictor of long-term mortality after CABG. Among the patients with NLR ≥ 3, MICS was associated with a significantly improved survival compared with sternotomy CABG.

## Background

Neutrophil lymphocyte ratio (NLR), a simple inflammatory marker, was found to be a significant independent predictor of adverse outcomes in patients with coronary artery disease [1-3]. The preoperative NLR was also a significant predictor of mortality among patients who underwent coronary artery bypass grafting surgery (CABG) [4]. Our goal was to evaluate the preoperative NLR as a predictor of mortality using our CABG population which includes both a conventional sternotomy CABG and the minimally invasive coronary artery bypass grafting (MICS) approach via left-thoracotomy [5]. We hypothesize that patients with elevated NLR, reflecting a higher preoperative inflammatory burden, will have better survival with MICS in comparison to sternotomy CABG. We evaluated the impact of these two different surgical approaches in patients with elevated NLR.

## Methods

This is an observational study of a prospectively maintained database to explore the predictive value of NLR on long-term survival of 1,177 patients who underwent coronary bypass surgery at the Heart Institute of Staten Island University Hospital in Staten Island, New York, a population of 443,728, between January 2005 and December 2008. A total of 1126 patients were included in the study and followed through December 2010; mean follow-up was 49 ± 15.2 months, with a range of 21 to 70 months. The only inclusion criterion was a preoperative complete blood count. Exclusion criteria included: hematological proliferative diseases, active or chronic inflammatory or autoimmune diseases, steroid therapy, concomitant valve surgery or unavailable complete blood count with differential preoperatively. Fifty-one patients were excluded from the study: 22 patients with concomitant valve surgery, 6 patients with hematological disease, 14 on steroids and 9 who did not have complete blood count with differential pre-operatively. The primary end point, all cause five year mortality, was obtained from the electronic medical record and the social security death index. Differential leukocyte counts were obtained by the Coulter counter technique (Coulter Gen S Hematology Analyzer, Beckman Coulter Corp ®, Hialeah, Florida).

Trained physicians reviewed electronic medical records for potential confounders, including demographic variables, coronary artery disease risk factors, comorbidities, prescribed medications before surgery and medications at discharge, operative variables, laboratory values, left ventricular ejection fraction, New York Heart Association (NYHA) and Canadian Cardiovascular Society (CCS) class (Tables 1 and 2). We obtained left ventricular ejection fraction from echocardiography reports. Positive smoking status included both current and prior smokers. To study the baseline characteristics according to the NLR, the patients were divided into equal tertiles according to NLR: 1^st^ tertile (NLR <2.2 with 371 patients), 2^nd^ tertile (2.2 ≤ NLR ≤ 3.4 with 375 patients), and 3^rd^ tertile (NLR >3.4 with 380 patients). The study was powered to evaluate the primary outcome with an estimated 7% 5-year mortality (7% versus 14%) difference between patients in the 1^st^ and 3^rd^ NLR tertiles. To achieve this difference with an 80% power and a 5% type 1 error required a minimum of 305 patients in each NLR tertile.

**Table 1:** Baseline characteristics (demographic and presentation) of coronary artery bypass patients according to neutrophil lymphocyte ratios (NLR) and surgical approach (MICS vs. Sternotomy CABG)

**Variables**	**NLR <2.3 (N = 371)**		***p *Value**	**2.3 ≤ NLR ≤ 3.4 (N = 375)**		***p *Value**	**NLR >3.4 (N = 380)**		***p *Value**
	**Sternotomy CABG**	**MICS**		**Sternotomy CABG**	**MICS**		**Sternotomy CABG**	**MICS**	
n	242	129		245	130		243	137	
Age (years)	62.5 ± 10.2	60.7 ± 10.9	0.12	64.3 ± 11.0	62.6 ± 10.7	0.14	67.5 ± 11.0	65.6 ± 10.7	0.10
Male	174 (71.9%)	90 (69.8%)	0.67	172 (70.5%)	93 (71.0%)	0.92	178 (73.3%)	100 (73.0%)	0.96
Race (Caucasian)	200 (82.6%)	106 (82.2%)	0.09	218 (89.3%)	123 (93.9%)	0.14	227 (93.4%)	128 (93.4%)	1.00
Body Mass Index (kg/m^2^)	29.1 ± 5.09	29.4 ± 5.42	0.65	30.0 ± 5.70	29.9 ± 6.22	0.95	28.8 ± 5.50	28.7 ± 5.71	0.81
Death	21 (8.7%)	9 (7.0%)	0.57	32 (13.1%)	17 (13.0%)	0.97	57 (23.5%)	18 (13.1%)	0.02
Family history of CAD	101 (41.7%)	63 (48.8%)	0.19	79 (32.4%)	45 (34.4%)	0.7	89 (36.6%)	41 (29.9%)	0.19
Smoking	149 (62.9%)	65 (50.4%)	0.02	141 (59.7%)	72 (55.4%)	0.42	134 (55.6%)	69 (50.4%)	0.33
Hypertension	172 (71.1%)	98 (76.6%)	0.26	180 (74.1%)	93 (71.0%)	0.52	181 (74.5%)	113 (83.1%)	0.05
Diabetes mellitus	94 (38.8%)	33 (25.6%)	0.01	73 (29.9%)	33 (25.2%)	0.33	96 (39.5%)	47 (34.3%)	0.32
Peripheral arterial disease	29 (12.0%)	13 (10.1%)	0.58	36 (14.8%)	7 (5.3%)	0.006	39 (16.0%)	14 (10.2%)	0.12
Renal failure on dialysis	3 (1.2%)	1 (0.8%)	0.68	3 (1.2%)	5 (3.8%)	0.1	8 (3.3%)	9 (6.6%)	0.14
Myocardial infarction	134 (55.4%)	51 (39.5%)	0.004	132 (54.1%)	52 (39.7%)	0.008	130 (53.5%)	38 (27.7%)	<0.001
History of PCI	71 (29.3%)	23 (17.8%)	0.02	66 (27.0%)	31 (23.7%)	0.48	58 (23.9%)	23 (16.8%)	0.11
Prior CABG	7 (2.9%)	2 (1.6%)	0.51	7 (2.9%)	1 (0.8%)	0.27	9 (3.7%)	2 (1.5%)	0.34
Prior heart failure	37 (15.3%)	7 (5.4%)	0.005	41 (16.8%)	9 (6.9%)	0.007	45 (18.5%)	11 (8.0%)	0.01
Prior cerebrovascular event	31 (12.8%)	8 (6.2%)	0.048	39 (16.0%)	11 (8.4%)	0.04	50 (20.6%)	12 (8.8%)	0.003
C0PD	30 (12.4%)	10 (7.9%)	0.18	34 (13.9%)	16 (12.2%)	0.64	34 (14.0%)	16 (11.7%)	0.52
Preoperative CCS									
none	0 (0%)	1 (0.8%)	0.14	2 (0.8%)	0 (0%)	0.02	2 (0.8%)	0 (0%)	<0.001
Class I	42 (17.6%)	24 (18.6%)		34 (13.9%)	32 (24.4%)		41 (17.2%)	28 (20.6%)	
Class II	61 (25.5%)	45 (34.9%)		74 (30.3%)	47 (35.9%)		46 (19.3%)	49 (36.0%)	
Class III	68 (28.5%)	33 (25.6%)		75 (30.7%)	29 (22.1%)		83 (34.9%)	45 (33.1%)	
Class IV	68 (28.5%)	26 (20.2%)		59 (24.2%)	23 (17.6%)		66 (27.7%)	14 (10.3%)	
NYHA									
Class I	22 (17.9%)	21 (19.4%)	0.21	25 (29.2%)	25 (24.0%)	0.31	30 (23.8%)	22 (21.2%)	0.03
Class II	33 (26.8%)	40 (37.0%)		42 (32.3%)	39 (37.5%)		26 (20.6%)	33 (31.7%)	
Class III	44 (35.8%)	26 (24.1%)		37 (28.5%)	19 (18.3%)		38 (30.2%)	37 (35.6%)	
Class IV	24 (19.5%)	21 (19.4%)		26 (20.0%)	21 (20.2%)		32 (25.4%)	12 (11.5%)	
Ejection Fraction (%)	39.8 ± 12.1	41.0 ± 10.8	0.32	39.1 ± 12.1	41.6 ± 10.4	0.04	39.3 ± 12.8	41.2 ± 12.4	0.17
Preoperative laboratory values									
Leukocyte (k/cc)	7.42 ± 2.31	7.20 ± 2.03	0.37	8.09 ± 2.22	7.61 ± 2.20	0.05	9.70 ± 3.13	8.87 ± 2.71	0.01
Neutrophils (k/cc)	4.06 ± 1.35	3.94 ± 1.29	0.40	5.28 ± 1.55	4.97 ± 1.51	0.06	7.45 ± 2.82	6.76 ± 2.45	0.01
Lymphocytes (k/cc)	2.45 ± 1.10	2.40 ± 0.763	0.40	1.91 ± 0.566	1.79 ± 0.533	0.06	1.35 ± 0.462	1.29 ± 0.455	0.24
Monocytes (k/cc)	0.62 ± 0.286	0.62 ± 0.241	0.80	0.65 ± 0.263	0.60 ± 0.225	0.12	0.69 ± 0.299	0.62 ± 0.237	0.02
Creatinine (mg/dl)	1.06 ± 0.741	1.07 ± 0.983	0.99	1.14 ± 0.711	1.19 ± 1.02	0.56	1.26 ± 0.61	1.18 ± 0.604	0.06
Glucose (mg/dl)	124.0 ± 48.9	122.4 ± 52.4	0.76	126.1 ± 49.3	110.8 ± 41.6	0.002	136.2 ± 58.8	121.5 ± 46.3	0.01

*CABG*  coronary artery bypass grafting, *CAD*  coronary artery disease, *CCS*  Canadian Cardiovascular Society Angina classification system, *COPD*  chronic obstructive pulmonary disease, *NYHA*  New York Heart Association Functional Classification, *PCI*  percutaneous coronary intervention. Categorical variables were presented as frequencies and percentages; continuous variables were presented as means and standard deviations.

**Table 2:** Operative and post-operative characteristics (management and laboratory) of coronary artery bypass patients according to neutrophil lymphocyte ratios (NLR) and surgical approach (MICS vs. Sternotomy CABG)

**Variables**	**NLR <2.3 (N = 371)**		***p *Value**	**2.3 ≤ NLR ≤ 3.4 (N = 375)**		***p *Value**	**NLR >3.4 (N = 380)**		***p *Value**
	**Sternotomy CABG**	**MICS**		**Sternotomy CABG**	**MICS**		**Sternotomy CABG**	**MICS**	
**n**	242	129		245	130		243	137	
**In-hospital medication**									
Aspirin	238 (98.3%)	128 (99.2%)	0.66	234 (95.9%)	130 (100%)	0.02	237 (97.5%)	137 (100%)	0.09
Clopidogrel	110 (45.5%)	111 (86.0%)	<0.001	128 (52.5%)	115 (88.5%)	<0.001	120 (49.4%)	116 (84.7%)	<0.001
Beta-blockers	232 (95.9%)	124 (96.1%)	0.91	230 (94.3%)	128 (98.5%)	0.06	231 (95.1%)	135 (98.5%)	0.08
Angiotensin convertase inhibitor	84 (34.7%)	44 (34.1%)	0.91	96 (39.3%)	31 (23.8%)	0.00	104 (42.8%)	45 (32.8%)	0.06
Angiotensin receptor blocker	29 (12.0%)	13 (10.1%)	0.58	17 (7.0%)	13 (10.0%)	0.30	19 (7.8%)	17 (12.4%)	0.14
Warfarin	18 (7.4%)	7 (5.4%)	0.46	18 (7.4%)	8 (6.2%)	0.66	26 (10.7%)	9 (6.6%)	0.18
Statin	232 (95.9%)	127 (98.4%)	0.23	229 (93.9%)	126 (96.9%)	0.20	228 (93.8%)	132 (96.4%)	0.29
**Medication on discharge**									
Aspirin	237 (98.3%)	125 (97.7%)	0.70	234 (95.9%)	127 (97.7%)	0.56	230 (95.4%)	131 (97.8%)	0.26
Clopidogrel	66 (27.4%)	101 (78.9%)	<0.001	83 (34.0%)	105 (80.8%)	<0.001	64 (26.6%)	104 (77.6%)	<0.001
Beta-blockers	212 (88.0%)	114 (89.1%)	0.76	216 (88.5%)	117 (90.0%)	0.66	208 (86.3%)	123 (91.8%)	0.11
Angiotensin convertase inhibitor	70 (29.0%)	32 (25.0%)	0.41	67 (27.5%)	24 (18.5%)	0.05	75 (31.1%)	27 (20.1%)	0.02
Angiotensin receptor blocker	1 (0.4%)	4 (3.1%)	0.05	4 (1.7%)	1 (0.8%)	0.66	2 (0.8%)	7 (5.2%)	0.01
Warfarin	30 (12.4%)	17 (13.3%)	0.82	27 (11.1%)	12 (9.2%)	0.58	43 (17.8%)	11 (8.2%)	0.01
Statin	220 (91.3%)	119 (93.0%)	0.57	222 (91.0%)	120 (92.3%)	0.66	218 (90.5%)	123 (91.8%)	0.67
**Operative data**									
Internal mammary artery utilized	231 (95.5%)	127 (98.4%)	0.23	230 (94.3%)	128 (97.7%)	0.13	221 (90.9%)	134 (97.8%)	0.01
Number of conduits used for bypass	3.28 ± 0.991	2.12 ± 0.673	<0.001	3.26 ± 0.886	2.28 ± 0.806	<0.001	3.12 ± 0.910	2.17 ± 0.753	<0.001
Cardiopulmonary bypass	27 (11.2%)	6 (4.7%)	0.04	30 (12.3%)	7 (5.3%)	0.03	18 (7.4%)	8 (5.8%)	0.56
**Postoperative complications**									
Renal Failure on Dialysis	1 (0.4%)	2 (1.6%)	0.28	3 (1.2%)	4 (3.1%)	0.25	12 (4.9%)	3 (2.2%)	0.19
New onset atrial fibrillation	33 (13.6%)	22 (17.1%)	0.38	40 (16.4%)	30 (22.9%)	0.12	48 (19.8%)	43 (31.4%)	0.01
Septicemia	0 (0%)	5 (3.9%)	0.005	0 (0%)	2 (1.5%)	0.12	6 (2.5%)	2 (1.5%)	0.72

Categorical variables were presented as frequencies and percentages; continuous variables were presented as means and standard deviations. *CABG*  coronary artery bypass grafting.

The NLR tertiles were analyzed using the chi-square test for categorical variables and Kruskall-Wallis test for continuous variables. The distributions of continuous and categorical variables were represented as mean ± standard deviation, frequencies and percentages, respectively. A univariate screen of all potential predictors of mortality using a separate Cox proportional hazards model for each variable was performed. Those variables that were found to be statistically significant (p < 0.05) as well as clinically meaningful in the univariate analyses were included in a multivariate Cox regression model. Backwards selection with significance level for removal from the model set at 0.1 was used to remove variables which did not significantly contribute information to this model, given other factors included in the model.

In order to avoid collinearity preoperative neutrophils and lymphocytes were not included in the model with preoperative NLR. The analysis of mortality was accomplished by applying standard methods of survival analysis (i.e., computing the Kaplan-Meier product limit curves where the data was stratified by NLR tertiles). In cases where the endpoint event (death) had not yet occurred the number of months until last follow-up was used and considered censored. The three NLR groups were compared using the log-rank test. Additionally, among elevated NLR group, a multivariate logistic regression was performed to find the best model that fits the data and that explains the association between mortality and several independent variables deemed either to have significant association with mortality as a binary response or to be considered as clinically important. A backward selection procedure, with significance level for removal from the model set at 0.1 was used. The odds ratios (OR) and 95% confidence interval (CI) of the best fitted model were computed. Two models were conducted in this multivariate stage. All analyses were performed using SPSS version 19.0 (SPSS Inc., Chicago, IL, USA). This study was approved by the institutional review board at Staten Island University Hospital.

## Results

Out of the 1126 patients included in the study, 1030 (91%) patients underwent off-pump CABG (MICS, 376/397 = 95%; Sternotomy CABG, 654/729 = 90%). The patients in the highest NLR tertile were significantly older with more Caucasian individuals in comparison to the lowest NLR tertile (p < 0.0001). Additionally, the patients in the highest NLR tertile had significantly higher preoperative serum creatinine and glucose levels with a higher prevalence of diabetes, preoperative end stage renal disease requiring hemodialysis and family history of coronary artery disease compared to those in the lowest NLR tertile. The three NLR tertiles had similar distribution of both preoperative NYHA and CCS classes. Postoperatively, the patients in the highest NLR tertiles suffered a significantly higher rate of renal failure requiring hemodialysis and new onset atrial fibrillation in comparison to those in the lowest NLR tertile.

**Figure 1:**
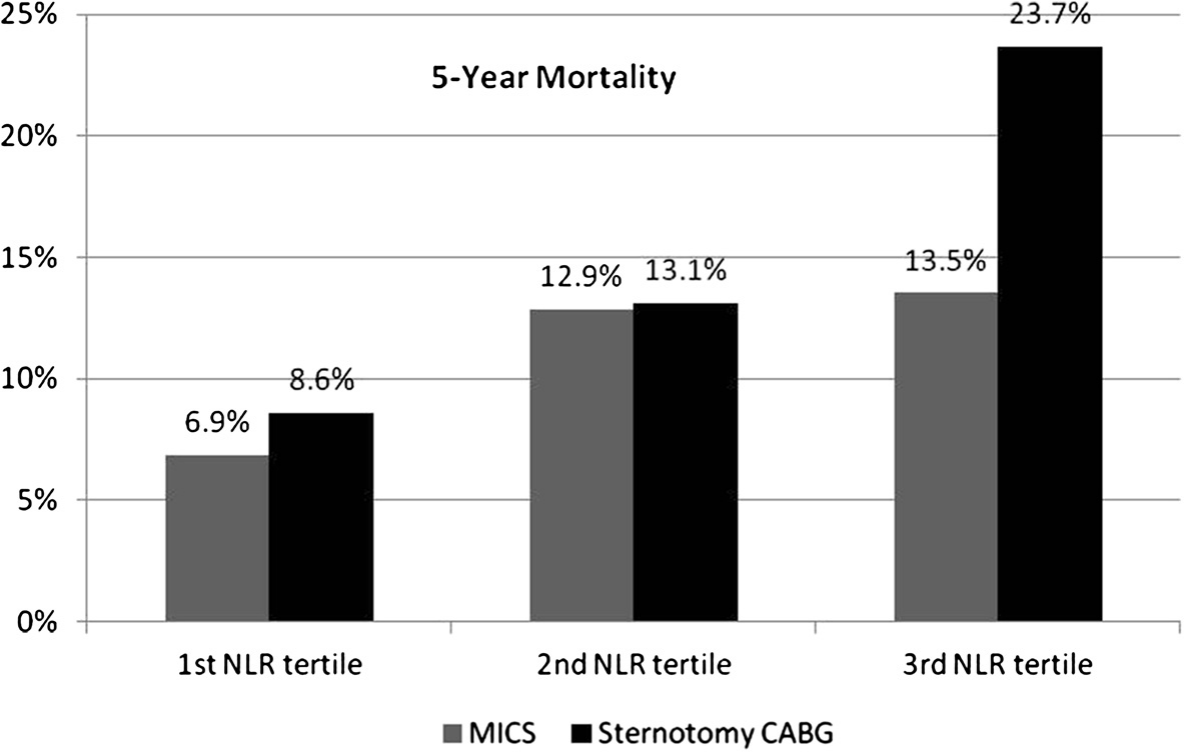
**The 5-year all-cause mortality after coronary bypass surgery according to preoperative neutrophil lymphocyte ratio (NLR) and surgical approach(MICS vs. Sternotomy CABG).** According to Fisher’s exact test the 5-year mortality rates were higher in the sternotomy CABG patients with NLR >3.4 compared to MICS with NLR >3.4 (p value <0.0001 ). MICS = minimal invasive cardiac surgery; CABG = coronary artery bypass grafting. The average follow-up was 49 ± 15.2 months (range 21–70 months). Total of 467 patients had 5-year follow up period.

**Figure 2:**
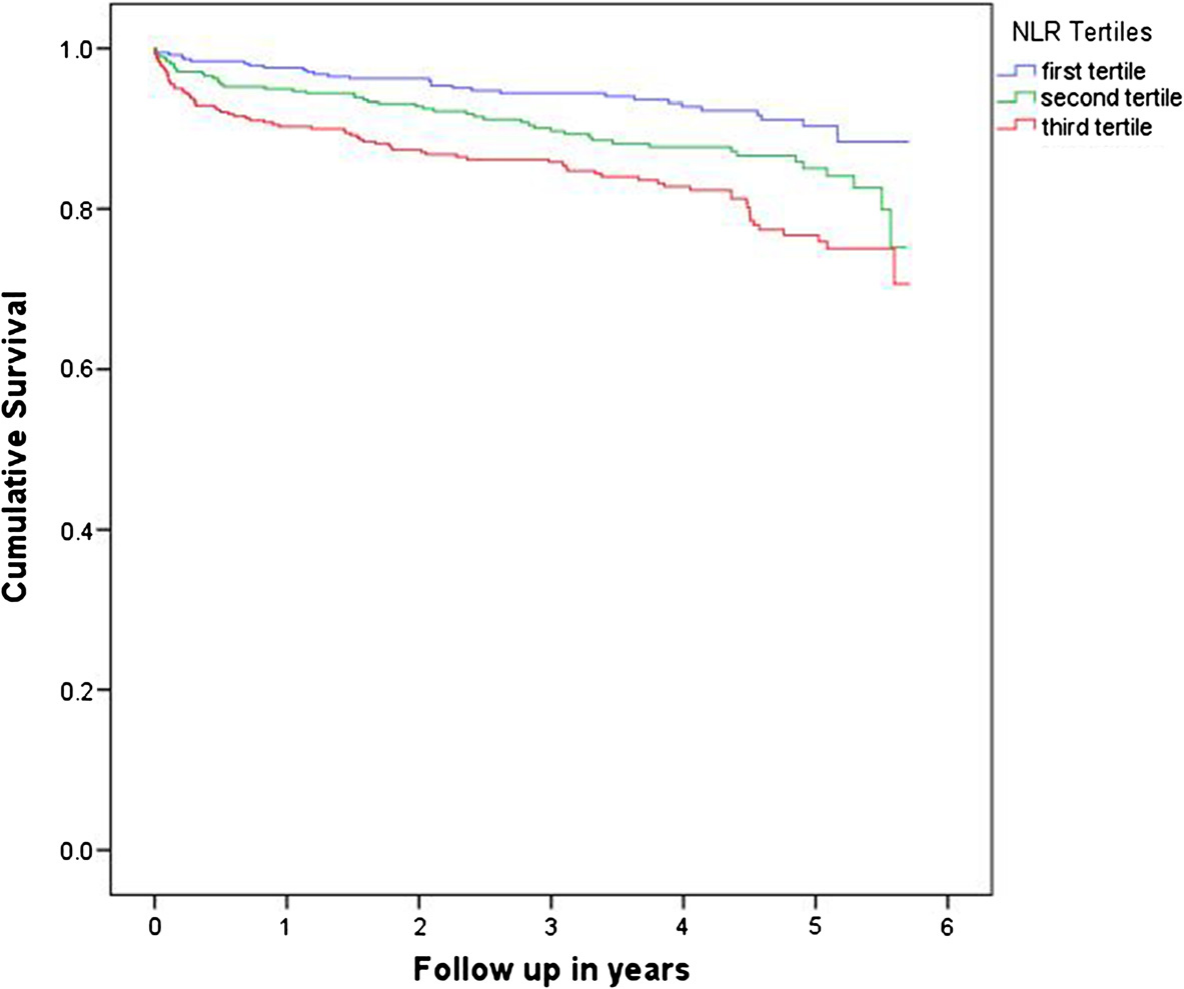
**Kaplan-Meier Curve of mortality among patients undergoing coronary artery bypass surgery according to their neutrophil lymphocyte ratio (NLR) tertiles.** Log rank (Mantel-Cox) p < 0.0001. The average follow-up was 49 ± 15.2 months (range 21–70 months). Total of 467 patients had 5-year follow up period.

The patients in the lowest NLR tertile had significantly lower 30-day, 6-month, 1-year and 5-year mortality rates (0.5%, 1.6%, 2.4% and 8%) in comparison to the middle NLR tertile (1.3%, 4.3%, 5.1% and 13%) and the highest NLR tertile (2.7%, 7.7%, 9.9% and 20%), respectively with p < 0.0001. When we compared the overall survival among MICS and sternotomy subgroups in each NLR tertile, the MICS had significantly lower mortality only among the highest NLR tertile (p value <0.001 according to two tailed Fisher’s exact test) (Figure 1). Similarly, Kaplan-Meier curves showed significant difference (Log rank [Mantel-Cox] p < 0.0001) mortality in the highest NLR tertile (NLR >3.4) compared to the middle (NLR 2.3-3.4) and the lowest (NLR <2.3) NLR tertiles (Figure 2). Table 3 demonstrates the univariate screening analysis (the individual Cox regressions examining the association between each variable and overall survival). In our study population, the variables associated with increased mortality were age, chronic pulmonary disease, congestive heart failure, prior cerebrovascular event, diabetes, family history of coronary artery disease, pre- and post-operative renal failure requiring dialysis, intraoperative use of cardiopulmonary bypass (CPB), elevated preoperative neutrophil, NLR, serum creatinine, serum glucose, postoperative septicemia, and new onset atrial fibrillation (Table 3). Male gender, use of internal mammary artery and higher lymphocyte count were associated with lower mortality. Elevated WBC, neutrophil, NLR and lower lymphocyte count were significant predictors of mortality in the univariate screening models. In the multivariate Cox proportional hazard models, including the confounding variables, NLR was the only independent predictor of long-term mortality (HR per unit increase in NLR was 1.06, CI 95% was 1.014-1.099, p = 0.008) (Table 4).

**Table 3:** Hazard ratios of baseline characteristics for all-cause mortality among coronary artery bypass surgery patients (univariate analysis)

**Variable**	**Hazard ratio (95% confidence interval)**	***p *Value**
Male	0.63 (0.45–0.87)	0.005
Age (per year)	1.06 (1.06–1.09)	<0.001
Race (Caucasian)	1.40 (0.78-2.53)	0.26
Body Mass Index (kg/m^2^) (31+)		
18–25	1.35 (0.90–2.03)	0.14
26–30	1.11 (0.75–1.64)	0.60
Ejection fraction (%)	1.01 (0.99–1.02)	0.46
Hypertension	1.31 (0.89–1.93)	0.17
Smoking	0.89 (0.65–1.23)	0.48
Chronic pulmonary disease	2.02 (1.37–2.96)	<0.001
Prior myocardial infarction	1.00 (0.73–1.37)	0.99
Prior coronary angioplasty	1.16 (0.81–1.65)	0.41
Prior coronary artery bypass graft	0.67 (0.21–2.09)	0.48
Congestive heart failure	1.64 (1.11–2.43)	0.01
Prior cerebrovascular event	1.79 (1.22–2.62)	0.003
Family history of coronary artery disease	0.56 (0.39–0.80)	0.001
Renal Failure on Dialysis	3.95 (2.19–7.12)	<0.001
Diabetes Mellitus	1.60 (1.17–2.21)	0.004
Peripheral arterial disease	1.39 (0.91–2.14)	0.12
Pre-Operative CCS	1.01 (0.87–1.18)	0.85
Pre-Operative NYHA	0.86 (0.71–1.06)	0.15
Pre-Operative Leukocyte	1.05 (0.99–1.11)	0.11
Pre-Operative Neutrophils	1.08 (1.02–1.15)	0.01
Pre-Operative Lymphocytes	0.69 (0.55–0.88)	0.003
Pre-Operative Monocytes	1.46 (0.91–2.36)	0.11
Pre-Operative Creatinine	1.20 (1.09–1.31)	<0.001
Pre-Operative Glucose	1.00 (1.00–1.01)	<0.001
Pre-Operative NLR	1.09 (1.06–1.13)	<0.001
**Operative and postoperative data**		
Internal mammary artery used	0.35 (0.21–0.57)	<0.001
Number of conduits used for bypass	0.96 (0.82–1.13)	0.64
Renal failure on dialysis	6.80 (3.91–11.80)	<0.001
Cardiopulmonary bypass	1.76 (1.11–2.79)	0.01
New onset atrial fibrillation	2.03 (1.41–2.91)	<0.001
Septicemia	6.95 (3.54–13.65)	<0.001

*CCS* Canadian Cardiovascular Society Angina classification system, *NLR* neutrophil lymphocyte ratio, *NYHA*  New York Heart Association Functional Classification.

**Table 4:** NLR multivariate adjusted all-cause mortality Cox proportional hazard ratio in coronary bypass surgery patients

**Variables**	**HR**	**95% CI**	***p *Value**
Pre-Operative NLR (per unit)	1.06	1.01–1.10	0.008
Age (per year)	1.07	1.05–1.09	<0.001
Male gender	0.73	0.51–1.03	0.06
Family history of coronary artery disease	0.71	0.49–1.04	0.07
Smoking	1.37	0.96–1.95	0.08
Preoperative renal failure on dialysis	5.56	2.98–10.4	<0.001
Chronic pulmonary disease	1.73	1.15–2.59	0.008
In-hospital angiotensin convertase inhibitor	1.41	1.00–1.99	0.05
In-hospital statin	0.37	0.20–0.67	0.001
Aspirin on discharge	0.29	0.15–0.56	<0.001
Postoperative renal failure on dialysis	2.34	1.16–4.71	0.01
Postoperative septicemia	2.99	1.29–6.93	0.01
Preoperative Glucose (pre mg/dl)	1.01	1.00–1.01	<0.001

*NLR*  neutrophil lymphocyte ratio, *HR*  hazard ratio, *CI*  confidence interval.

**Figure 3:**
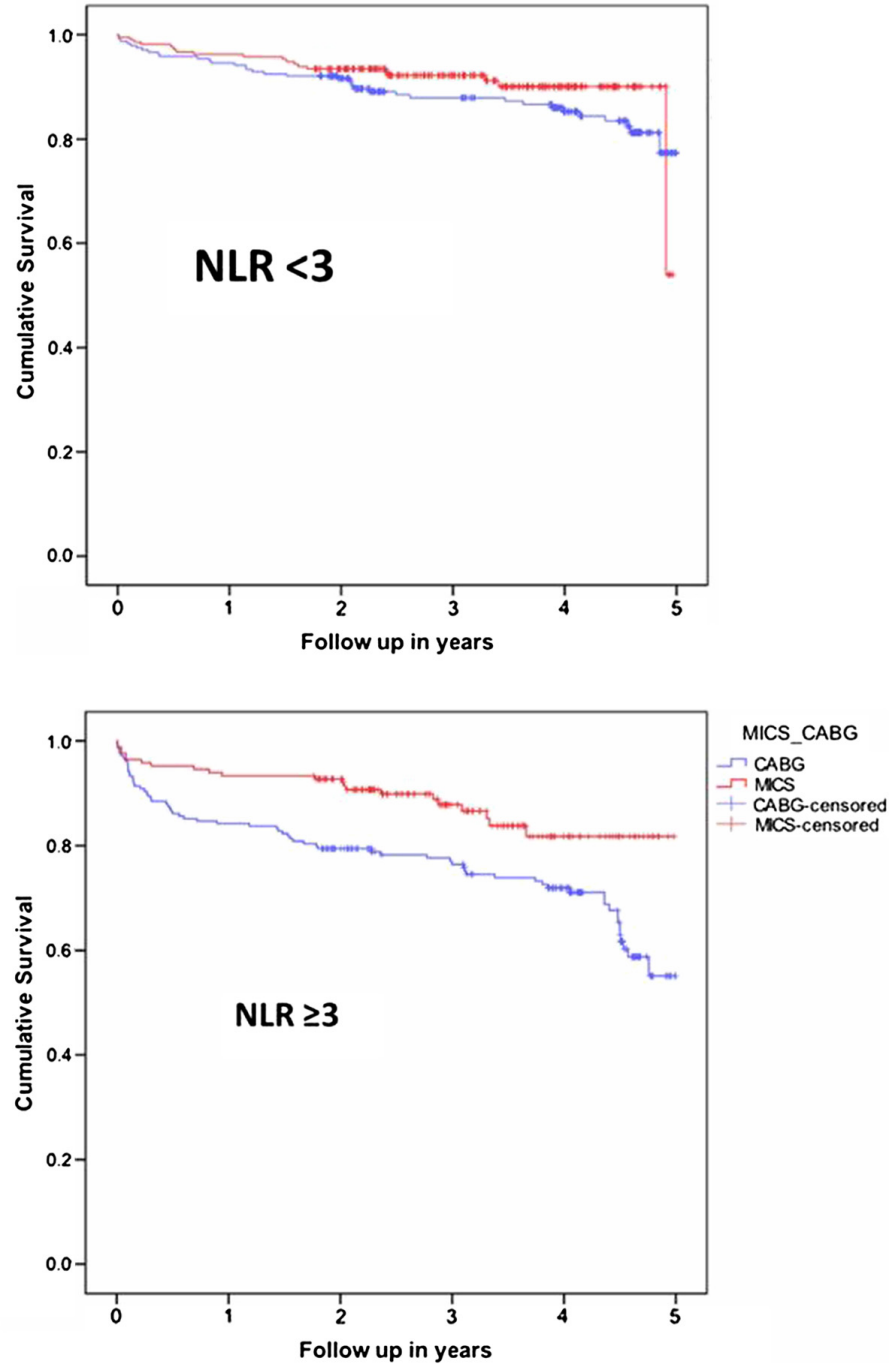
**Kaplan-Meier Curve of mortality among patients undergoing coronary bypass surgery according to their neutrophil lymphocyte ratio (NLR) cutoffs: NLR <3 and NLR ≥3 (p = 0.002).** The average follow-up was 49 ± 15.2 months (range 21–70 months). Total of 467 patients had 5-year follow up period.

When patients were divided into two groups according to the median NLR (NLR = 3) of the whole population, we found 624 patients with NLR <3 and 502 with NLR ≥3. Among the patients with NLR <3, there was no significant difference in the overall survival between the MICS and sternotomy CABG (21/221 = 9.5% vs. 40/403 = 9.9%, p = 1). Contrarily, the patients with NLR ≥ 3 who underwent MICS had a significantly lower mortality compared to sternotomy CABG (23/176 = 13.1% vs. 70/326 = 21.5%, p = 0.022). Kaplan-Meier curves showed significant difference in (Log rank [Mantel-Cox] p < 0.002) mortality between the MICS and sternotomy CABG with NLR ≥ 3 compared to NLR <3, p = 0.002 (Figure 3). Additionally, the patients with NLR ≥ 3 who underwent MICS had a significantly lower mortality (20/166 = 12% vs. 63/293 = 21.5%, p =0.011) compared to those who underwent off-pump sternotomy CABG, after excluding MICS patients who underwent CPB. Among the patients with NLR ≥ 3, multivariate regression models demonstrated that MICS CABG decreased the odd ratios of all-cause mortality by 56% and 50% according to model A and B, respectively (Table 5), compared to sternotomy.

**Table 5:** Multivariate logistic regression models for all-cause mortality among patients with preoperative neutrophil lymphocyte ratio ≥3

**Variable**	**Odd ratio**	**95% confidence interval**	***p *Value**
**Model A**			
MICS vs. Sternotomy CABG	0.44	0.24–0.78	0.005
Internal mammary artery used	0.39	0.17–0.89	0.02
Postoperative new onset atrial fibrillation	1.72	1.01–2.93	0.04
Beta blockers on discharge	0.57	0.30–1.09	0.09
In-hospital clopidogrel	1.56	0.92–2.63	0.09
**Model B**			
MICS vs. Sternotomy CABG	0.50	0.28–0.90	0.02
Age (per year)	1.08	1.05–1.11	<0.001
Preoperative renal failure on dialysis	10.05	3.46–29.19	<0.001
Internal mammary artery used	0.48	0.20–1.12	0.08

Model A included history of prior coronary angioplasty, MICS vs. sternotomy CABG, postoperative new onset atrial fibrillation, use of internal mammary artery, use of cardiopulmonary bypass, clopidogrel on discharge and in-hospital, coumadin on discharge, beta blockers on discharge.

Model B included age, gender, family history of coronary artery disease, history of renal dialysis, statin on discharge, coumadin on discharge, use of internal mammary artery, MICS vs. sternotomy CABG and postoperative new onset atrial fibrillation.

*MICS*  minimal invasive cardiac surgery (thoracotomy), *CABG*  coronary artery bypass graft.

## Discussion

The principle finding of our study is that elevated preoperative NLR independently predicts long term mortality in patients undergoing coronary artery bypass surgery via minimally invasive and sternotomy approaches. Those with elevated preoperative NLR ≥3 had better survival with MICS than with sternotomy CABG after adjusting for confounding variables. Elevated NLR was associated with a higher prevalence of diabetes, renal failure, and elevated serum creatinine and glucose. This association concurs with existing literature that describes these conditions as proinflammatory [6,7]. Similar to a prior study, we have observed an association between elevated preoperative NLR and the development of postoperative new onset atrial fibrillation [4].

Similar to prior studies, we found that NLR is superior to WBC, neutrophil and lymphocyte counts in predicting mortality. Because it is a ratio, NLR is relatively more stable than individual leukocytic parameters that are easily altered by many simple conditions (e.g. dehydration, over-hydration, diluted blood specimens, in-vitro blood specimen handling). In the NLR, the numerator is the neutrophil count (a higher neutrophil count is associated with an increased risk of death) and the denominator is the lymphocyte (a lower lymphocyte count is associated with an increased risk of death). This makes the NLR an augmented predictor of mortality. We hypothesize that an elevated neutrophil count is a marker for an active inflammatory process and a low lymphocyte count is a marker of failure to control this active inflammatory process. Many studies have demonstrated that neutrophils play a major role in atherosclerosis and plaque instability through various mediators such as neutrophil gelatinase-associated lipocalin (NGAL), neutrophil-elastase, myeloperoxidase, defensins and cathelicidin [8-11]. Studies have also demonstrated that preoperative statin therapy increases neutrophil apoptosis and reduces neutrophil-endothelial adhesion after cardiac surgery. These desirable statin effects were irrespective of lipid lowering action [12,13].

We hypothesize that a reduced peripheral blood lymphocyte count acts as a marker for an ongoing nonspecific atherosclerotic inflammatory process. Lymphopenia has been shown to be associated with atherosclerosis progression and major adverse cardiac outcomes [14,15]. Lymphocytes modulate smooth muscle proliferation via interferon gamma [16] which controls the course of luminal narrowing in atherosclerosis.

Prior studies evaluated CPB as a confounding variable to the inflammatory response after MICS versus sternotomy CABG. Gu et al. demonstrated in a randomized controlled trial (31 patients in each arm) that anterolateral thorocotomy was associated with a lower inflammatory response (leukocyte elastase, platelet β-thromboglobulin, and complement C) than sternotomy CABG [17]. However some of the sternotomy CABG patients in this study underwent CPB, which is believed by many to contribute greatly to the postoperative inflammatory response. According to our multivariate analysis, NLR predicted mortality after adjusting for CPB. Similarly in patients with NLR >3, MICS had better survival than sternotomy CABG after adjusting for CPB. Moreover, among patients with NLR >3, MICS off-pump had a significant lower mortality compared to off-pump sternotomy CABG. These results demonstrate that CPB was not a confounding variable to the superiority of MICS in these patients with elevated inflammatory marker. However, our study included only 96 on-pump CABG patients, which requires further studies with larger number of on-pump patients.

We compared the inflammatory response between MICS and sternotomy CABG. Gu et al. prospectively studied the inflammatory response in patients that underwent CABG without CPB. They found the inflammatory response in patients without CPB was lower in the anterolateral thoracotomy group compared to the sternotomy group [18]. In a randomized controlled trial of 30 patients, Diegeler et al. studied various inflammatory markers in three groups of CABG: sternotomy CABG with CPB, off-pump sternotomy CABG and limited anterior thoracotomy CABG approaches. The anterior thoracotomy approach had a lower interleukin 8 (pro-inflammatory marker) and a higher interleukin 10 (anti-inflammatory marker) than the off-pump sternotomy CABG [19]. The complement 3d, TNF receptor p55 and p75 showed a significant increase in both on and off-pump sternotomy CABG, but no change from the anterior thoracotomy group [19]. In a study of patients that underwent lung volume reduction surgery, bilateral thoracoscopic surgery had reduced inflammatory cytokines compared to sternotomy [20]. Likewise, in an experimental study using animals that underwent CPB with different surgical approaches, avoidance of full-sternotomy was associated with a reduced level of cytokines and inflammatory response. The animals without sternotomy had the lowest pro-inflammatory (IL-6 and IL-8) cytokines compared to mini and full sternotomy [21].

The superiority of MICS to sternotomy CABG in the group with high NLR may be related to thymus dysfunction after sternotomy. In a case–control pediatric study, 29% of children with sternotomy after cardiac surgery had an identifiable thymus on 14-month magnetic resonance image compared to 92% in the control group who had no cardiac surgery. The authors suggested that postoperative residual thymic tissue does not regenerate [22]. Additionally, in a study that included pediatric and adult populations, cardiothoracic surgery and incidental thymectomy were associated with immune dysregulation and unclear factors that altered the regulatory T cells [23]. In adult patients, thymectomies were followed by immune dysregulation and development of autoimmune diseases [24,25].

### Limitations

This is a single center study with patients who were retrospectively enrolled from our database. We have not included the operating surgeon in our data, which could be a possible confounding variable. The MICS patients with high NLR had a higher rate of internal mammary artery use compared to the sternotomy group with high NLR. Although the internal mammary artery use was included in the multivariate analysis, randomized prospective studies are needed to alleviate such significant difference in operative variables. We used all-cause mortality rather than cardiac related mortality. We also did not obtain data regarding other major cardiovascular adverse events during the follow-up period (e.g. myocardial infarction, stroke and later need for revascularization). There is the possibility of missing a number of primary outcomes by using the social security death index and medical records to assess the survival status. The smoking data are missing exact duration of smoking or cessation. Another limitation to our study is the lack of measurement for known inflammatory markers such as interleukins, C-reactive protein and erythrocyte sedimentation rate. Moreover, we had mostly off-pump CABG patients (91%), which limits the generalizability of our findings.

## Conclusion

Elevated preoperative NLR is an independent predictor of long-term mortality after CABG. Patients with preoperative NLR ≥ 3 had a better 5-year overall survival in MICS versus sternotomy CABG. This effect was not attributed to cardiopulmonary bypass or any other confounding variables. Further studies are needed to explain the mechanism and evaluate therapeutic applications to these findings.

## Abbreviations

NLR: Neutrophil lymphocyte ratio; CABG: Coronary artery bypass grafting surgery; MICS: Minimally invasive coronary artery bypass surgery.

## Competing interests

The authors declare that they have no competing interests.

## Authors’ contributions

BA researched data, contributed to the discussion, and wrote the manuscript. JTM designed the protocol, contributed to the discussion, and reviewed/edited the manuscript. MAS collected, researched data, contributed to the discussion, and reviewed/edited the manuscript. RB analyzed and researched data. JPN contributed to the discussion and reviewed/edited the manuscript. All authors read and approved the final manuscript.

## Oral presentation

International Congress of Coronary Artery Disease (ICCAD), 2011 Venice, Italy.
